# Having a stimulating lifestyle is associated with maintenance of white matter integrity with age

**DOI:** 10.1007/s11682-021-00620-7

**Published:** 2022-01-17

**Authors:** Gabriel Ducharme-Laliberté, Samira Mellah, Sylvie Belleville

**Affiliations:** 1 Department of Psychology, Université de Montréal, Montreal, QC Canada; 2 Research Center of the Institut universitaire de gériatrie de Montréal (CRIUGM), CIUSSS du Centre-Sud-de-L’Île-de-Montréal, Montreal, QC Canada

**Keywords:** Reserve, Brain maintenance, Working memory, Aging, White matter lesions

## Abstract

Brain maintenance refers to the fact that some older adults experience few age-related changes in the brain, which helps maintain their cognition. The goal of this study was to assess maintenance of white matter integrity by testing whether reserve proxies, measuring factors associated to a stimulating lifestyle, affect the maintenance of white matter integrity. Another goal was to measure whether maintenance of white matter integrity explains inter-individual differences in working memory (WM). Forty-one cognitively healthy older adults received a structural magnetic resonance imaging (MRI) examination to measure white matter lesions. They completed an n-back WM task with different loads (1- & 2-back), along with a questionnaire on their lifestyle. There was a positive association between age and volume of white matter lesions. This association was no longer found in those with higher scores on reserve proxies. In addition**,** smaller volumes of white matter lesions were associated with better performance than expected for age in the 1-back WM task. Better WM is associated with the maintenance of white matter integrity in older adults, which in turn is linked to measures reflecting a stimulating lifestyle throughout life.

**Supplementary Information:**

The online version contains supplementary material available at 10.1007/s11682-021-00620-7.

## Introduction

*Brain maintenance* refers to the preservation of structural, neurochemical or functional integrity and the absence of age-related brain pathology with age (Cabeza et al., 2018; Nyberg et al., 2012; Stern et al., 2018). White matter lesions (WML) are among the most frequent neuropathological changes observed in older adults (Brickman et al., 2008; Launer, 2004; Raz et al., 2003; Sachdev et al., 2007). WML are more common in the frontal (Grajauskas et al., 2019) and parietal lobes (Kennedy & Raz, 2015), two regions that are critical for WM.

A large number of studies have shown that the accumulation of WML explains the effects of age on working memory (WM) and executive functions in older adults (Oosterman et al., 2008; Raz et al., 2007; Vannorsdall et al., 2009). While WM performance is known to decline with age (Reuter-Lorenz & Park, 2010; Sylvain-Roy et al., 2015), older adults experience a higher level of interindividual WM variability compared to younger people (Sylvain-Roy & Belleville, 2015). This increased interindividual WM variability may be explained by differences in maintenance of white matter integrity.

Differences in socio-behavioural characteristics that reflect a stimulating lifestyle, such as education, leisure activities, profession or differences in lifetime exposure to knowledge (e.g.: national adult reading test or vocabulary subtest of the WAIS), may also play a role in the observed association between WML and cognition. For this reason, these variables have been widely used as reserve proxies. Some studies have found that reserve proxies (Lövdén et al., 2010; Luk et al., 2011; Teipel et al., 2009; Wirth et al., 2014) are associated with improved white matter integrity in older adults, but other studies have reported inconsistent findings (Arenaza-Urquijo et al., 2011; Gold et al., 2013; Vaqué-Alcázar et al., 2017). This may be due to the use of different white matter integrity indicators (e.g., white matter hyperintensities vs. fractional anisotropy, etc.), statistical operationalization (e.g., whole-brain group differences vs. regions of interest) or types of proxies used (e.g. bilingualism or education vs. more inclusive variables). Thus, further work is needed to clarify the potential neuroprotective effects of cognitively stimulating lifestyles against age-related WML.

The main objective of the present study is to assess brain maintenance by measuring whether reserve proxies moderate the relationship between age and WML. Critically, we investigated the potential moderating effect of reserve proxies on the relationship between age and WML, rather than just controlling for its effect as has been done in previous studies. We then tested whether the volume of WML moderates the relationship between age and WM performance. This is important to measure cognitive resilience in older age. Indeed, few prior studies have considered the effects of reserve proxies on the relationship between WML and age-sensitive cognitive processes such as WM. If socio-behavioral characteristics and lifetime exposure to knowledge affect brain maintenance, we would expect to find less WML than predicted by age in participants who have higher scores on these reserve proxies. We would also expect older adults with lower volumes of WML to have better WM performance than expected given their age. These findings would support the notion that a stimulating lifestyle provided through socio-behavioural characteristics and a lifetime of exposure to knowledge preserve WM by reducing the volume of WML. To this end, we determined individual reserve proxy composite scores that were calculated by combining the scores from a socio-behavioural questionnaire and the WAIS vocabulary subtest. Moreover, each individual score was weighted according to the results of a principal component analysis (PCA). Finally, intracranial volume (ICV) will be considered as an estimate of premorbid brain volume to determine whether the effect of lifestyle is specific to the maintenance of white matter integrity. ICV will also be assessed to determine whether it increases resilience to the effects of age on WM and moderates the relationship between age and WM performance.

## Methods

### Participants

Forty-one healthy French-speaking older adults were recruited through the participants bank of the Research Center of the *Institut universitaire de gériatrie de Montréal* and advertisements posted in the community. This sample size allows sufficient power (0.80) to test the moderation term (two-tailed) in the regression model, assuming a medium effect size (*f*2 = 0.2) and an alpha value of *p* = 0.05. All participants were right-handed and had normal or corrected-to-normal vision and hearing. Participants received a thorough clinical and cognitive assessment, which included dementia screening tests and clinical questionnaires. The following established measures were used (Solé-Padullés et al., 2009): the fourth Wechsler Adult Intelligence Scale *Vocabulary* subtest score as an estimate of accumulated general knowledge, and the Cognitive Reserve Questionnaire (CRQ; (Rami et al., 2011)), which measures educational attainment, professional occupation and leisure activities throughout life.

Exclusion criteria were past or present neurological or cognitive disorders, alcoholism or substance abuse, severe psychiatric disorders, general anesthesia within the last six months, use of any neurotropic medication, as well as scores below the education or age/education adjusted cut-off respectively on dementia screening tests.

### Reserve proxy measures

We determined individual composite scores to reflect reserve proxies. Our goal was to provide a single score that would reflect a combination of typical reserve measures. Individual composite scores (sum of the weighted values) were created based on PCA, which were obtained using three scores from our reserve proxies: i.e., scores on the vocabulary subtest, the occupation-education subscale from the CRQ and the leisure subscale from the CRQ. The CRQ is a 15-item questionnaire measuring educational attainment and early childhood educational context (5 questions), professional occupation (1 question), intellectually stimulating leisure (4 questions), physical activities (2 questions) and socially stimulating activities during the course of the person’s life (3 questions).The occupation-education subscale comprises questions on educational attainment, early childhood educational context and professional occupation. The leisure subscale includes questions on intellectual, physical and social activities. The PCA was computed by forcing the three proxy measures of cognitive reserve mentioned above (i.e., scores on the vocabulary subtest and the CRQ subscales) into a single factor where each of these proxy measures were weighted in order to maximize the explained variance ((Stern et al., 2005); see supplementary material section for more detailed information on the PCA and resulting matrix (Table S1)). Individual composite scores for each participant were then automatically created by the PCA by summing up the weighted values obtained for these three variables.

### N-back working memory task

An n-back task was used to assess WM performance (Braver et al., 1997). In the n-back task, participants were presented lists of sequentially appearing letters and asked to indicate using a yes/no button whether the displayed letter matched the one shown in the 1-back or 2-back position. In the 0-back control condition, series of letters were displayed in the center of a screen and participants determined whether the letter was an "X". The three conditions (0-, 1- and 2- back conditions) were presented in 15 blocks, each containing 16 letters (five targets) alternating between the three conditions. Letters were presented at a rate of 500 ms per item with a 1500 ms crosshair interstimuli interval. A discrimination index or corrected hit rate (*hit rate* minus *false alarm rate*; H-FA) was calculated for each condition (Snodgrass & Corwin, 1988).

### Neuroimaging data

#### MRI acquisition parameters

Magnetic resonance imaging data were collected on a Siemens Magnetom Trio 3 T MRI system (Siemens Medical Solutions, Erlangen, Germany), using the Siemens 32-channel receive-only head coil at the Functional Neuroimaging Unit of the Research Center of the *Institut universitaire de gériatrie de Montréal*. A structural high-resolution T1-weighted 3D-Multi-Echo MPRAGE sequence (TR: 2530 ms; TE: 1.64 ms; flip angle: 7°; FoV: 256 mm; voxel size: 1.0 × 1.0 × 1.0 mm; 176 continuous slices) was acquired for volumetric analyses. A FLAIR weighted sequence (*Fluid Attenuated Inversion Recovery*; TR: 9000 ms; TE: 90 ms; flip angle: 150°; FoV: 240 mm; voxel size: 0.9 × 0.9 × 4.0 mm with 4.0 mm distance gap factor; 44 slices) was also obtained for WML confirmatory analysis.

#### Preprocessing and first-level analyses

Participants’ structural T1-weighted scans were analyzed through FreeSurfer 5.3 automated software (https://surfer.nmr.mgh.harvard.edu/). The procedure included automated geometric topology correction, inter-subject alignment and whole-brain volumetric segmentation – including white matter hypointensities and estimated intracranial volumes, which were of interest for the present study (Fischl, 2012). The process was reviewed slice-by-slice at each step, and tissue misclassification was manually adjusted when necessary.

WML obtained through the analysis with FreeSurfer were validated through a semi-automated technique developed by DeCarli et al. (2005) using FLAIR images (only available for 37 participants). After manual removal of non-brain tissues from the images and the automated removal of image artifacts, brain matter and cerebrospinal fluid were modeled in a semi-automated fashion. Voxels exceeding the default threshold were then characterized as white matter lesions. Scans were inspected slice by slice to ensure that segmentation only included WML and excluded any noise, and the threshold was manually adjusted when necessary. After obtaining a satisfactory mask of hyperintense voxels, WML volume was automatically calculated for each participant. A Pearson’s correlation was then performed with the two measures of WML (based on T1 and based on FLAIR). There was a very high degree of consistency between both measures (*r*(35) = 0.96, *p* < 0.001). Because there were some missing data for the FLAIR images, FreeSurfer values were used in subsequent analyses to increase power, after being proportioned according to the participant’s estimated intracranial volume (ICV). This last manipulation was done in order to account for variations in head size.

### Statistical analyses

All statistical analyses were computed with the IBM Statistical Package for Social Science 25 (SPSS) and results were interpreted if they reached a threshold of *p* < 0.05. Pearson’s, Spearman’s or point biserial correlations were computed between the composite lifestyle score and potential confounding variables.

#### Brain maintenance

Moderation analyses were computed to assess if the reserve proxy composite score moderated the effect of age on WML volume, and if WML volume moderated the negative effect of age on cognition. The terms of the interactions were respectively created by multiplying age with the composite score and WML volume (all variables were centered). A hierarchical multiple regression was conducted using the “age by composite” interaction term to predict WML volume. Two other hierarchical multiple regressions were also conducted using the “age by WML volume” interaction term to predict WM performance separately in the 1-back and 2-back conditions. When significant, the moderation effects were visualized and probed through the PROCESS macro for SPSS (www.processmacro.org) following the procedure described in Hayes and Rockwood (2017). In sum, based on the resulting regression equation, the macro automatically computes the predicted values (y) from different combinations of the predictor (x) and moderator variables (w), that is from low, middle and higher values (i.e., at the 16^th^, 50^th^ and 84^th^ percentile) included within the range of the original data. Then, to facilitate interpretation of the effect of the moderator (w) on the relationship between x and y, the computed values were plotted in a diagram allowing the graphic visualization of the equation-based regression slopes for the low, middle and higher values of the moderator (w). This is an important step to better understanding how the effects of x on y vary depending on the value of w (which is continuous instead of categorial as in a standard analysis of variance). The macro further generates a simple slope analysis, which provides the conditional effects of the predictor (x) on the predicted variable (y) and the significance levels for low, middle and high values of the moderator (i.e., at the 16^th^, 50^th^ and 84^th^ percentile). This approach maintains the continuous nature of the regression analysis and avoids splitting the original data into trivial categories, which would not respect the mathematical principles behind regression models and would prevent the slope equations to be driven only by a few artificially pulled together observations and that might vary depending on the arbitrary categorization (for a more detailed explanation of the rationale, see Hayes and Rockwood (2017)).

#### Effect of estimated premorbid brain volume

Moderation analyses assessed the interaction between ICV and the effect of age to predict WM performance. Two separate hierarchical multiple regressions were conducted using the interaction term between age and ICV (both variables were centered) to predict WM performance in the 1-back and 2-back conditions. Significant moderating effects were then visualized and probed as mentioned above.

## Results

Two participants among the forty-one initially recruited withdrew consent before the experimental session, one for health reasons and the second did not wish to have an MRI examination. The characteristics of the 39 remaining participants (24 women) are shown in Table 1. No correlation was found between the composite lifestyle score and age, sex, or cognitive and clinical measures. These variables were therefore not examined further in relation to composite lifestyle score.

**Table 1:** Demographic, clinical and behavioural characteristics of participants

	*M* (*SD*)	Range
Age	73.10 (5.64)	65.00 – 88.00
MoCA (/30)	28.51 (1.43)	24.00 – 30.00
MMSE (/30)	28.72 (1.17)	25.00 – 30.00
Stroop (plate 3; time)	26.87 (7.81)	15.30 – 47.81
RL/RI (total delayed recall; /16)	15.74 (0.55)	14.00 – 16.00
GDS (/15)	1.44 (1.67)	0.00 – 6.00
Charlson’s (/37)	0.49 (0.79)	0.00 – 2.00
Hachinski’s (/18)	0.54 (0.68)	0.00 – 3.00
ADL (/45)	0.76 (1.32)	0.00 – 5.00
N-back discrimination indexes (H-FA)		
1-back	0.85 (0.18)	0.33 – 1.00
2-back	0.70 (0.12)	0.30 – 0.92

Montreal Cognitive Assessment (MOCA; Nasreddine et al., 2005); Mini-Mental State Examination (MMSE; Folstein et al., 1975); Stroop-Victoria (Regard, 1981); Free and Cued Recall Test (RL/RI; Van der Linden, 2004); Geriatric Depression Scale (GDS; Yesavage et al., 1982); Charlson’s Comorbidity Index (Charlson et al., 1987); Hachinski’s Ischemic Score (Hachinski et al., 1975); Activities of Daily Living Inventory (Galasko et al., 1997)

### Brain maintenance

**Fig. 1:**
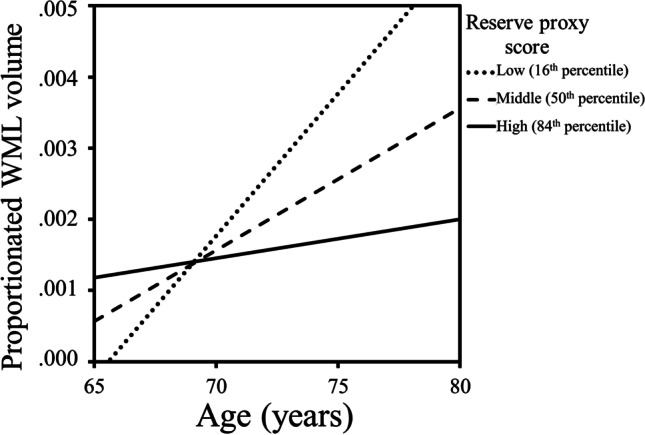
Diagram displaying regression slopes based on the predicted values (16^th^, 50^th^ and 84^th^ percentile) that resulted from the moderation analysis equation investigating the relationship between age and WML volume as a function of the level of engagement in a stimulating lifestyle

The regression model predicting WML volume (*F*(3, 35) = 6.70, *p* = 0.001) revealed a significant effect of age (β = 0.51, *p* = 0.001; *sr*^2^ = 0.24) and a marginally significant effect of the composite score (β = -0.26, *p* = 0.059). These two variables interacted (β = -0.33, *p* = 0.023; *sr*^2^ = 0.10) to predict WML volume. Figure 1 shows that the positive relationship between age and WML volume is not found in those with higher composite scores. Futhermore, the conditional effects of age on WML are only significant for the low (*T* = 3.71, *p* < 0.001) and middle (*T* = 3.43, *p* = 0.002) ranges of the composite score, and were non-significant for the higher range (*T* = 0,66, *p* = 0.511).

**Fig. 2:**
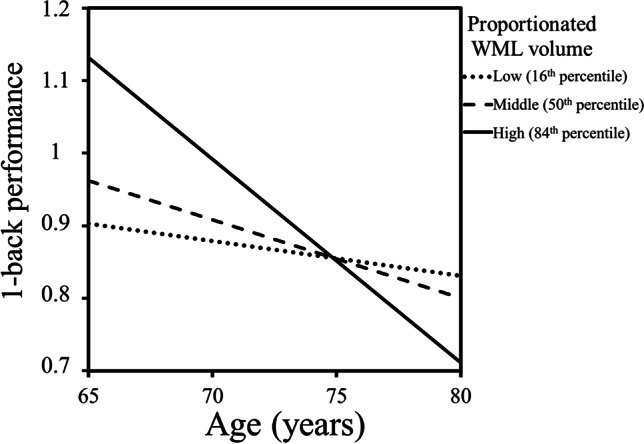
Diagram displaying regression slopes based on the predicted values (16^th^, 50^th^ and 84^th^ percentile) that resulted from the moderation analysis equation investigating the relationship between age and 1-back performance (Hits-False Alarms) as a function of the WML volume

The regression model predicting WM performance for the 1-back condition (*F*(3, 35) = 6.69, *p* = 0.001) revealed a significant effect of age (β = -0.49, *p* = 0.003; *sr*^2^ = 0.18) but no significant effect of WML alone (β = 0.20, *p* = 0.366). Importantly, age and WML volume interacted (β = -0.50, *p* = 0.014; *sr*^2^ = 0.12) to predict performance. Figure 2 shows a negative relationship between age and WM performance on the 1-back condition, which is weaker with lower WML volume. Conditional effects of age on WM performance were only significant for the high (*T* = 3.56, *p* = 0.001) and middle (*T* = 2.19, *p* = 0.035) range of WML volume, and non-significant for the lower (*T* = 0.85, *p* = 0.403) range. The regression model predicting WM performance in the 2-back condition was not significant (*F*(3, 35) = 2.08, *p* = 0.121) and thus not examined further.

### Effect of estimated premorbid brain volume

The regression models predicting WM performance based on premorbid brain volume were not significant for the 1-back (*F*(3, 35) = 2.43, *p* = 0.067) nor 2-back condition (*F*(3, 35) = 1.48, *p* = 0.230).

## Discussion

The goal of this study was to investigate whether having a stimulating lifestyle (measured using socio-behavioural characteristics and lifetime exposure to knowledge) helps older adults better maintain white matter integrity and WM capacities with age. Our focus was on WML, as these represent a prominent alteration found in the normal aging brain. WML are also associated with WM decline, a cognitive function that is crucial to numerous complex cognitive tasks. Results indicate that higher reserve proxies are associated with a smaller volume of WML than predicted by age, suggesting better maintenance of white matter integrity. Therefore, while older age is associated with higher volumes of WML, this is not the case for people who have engaged in a more stimulating lifestyle.

The finding that a cognitively stimulating lifestyle has a protective impact on WML is consistent with a few prior studies (Luk et al., 2011; Teipel et al., 2009; Wirth et al., 2014). White matter microstructure properties have also been shown to be modifiable through cognitive training, even in older age (Lövdén et al., 2010). However, other inconsistent studies have shown that cognitively stimulating lifestyles have a negative impact on white matter integrity in healthy older adults (Arenaza-Urquijo et al., 2011; Gold et al., 2013; Vaqué-Alcázar et al., 2017). This counterintuitive finding was interpreted as reflecting a better tolerance: stimulating lifestyles may help older adults remain cognitively intact despite the presence of WML. Inconsistent results may also be due to the way proxies were measured across studies or to differences in the vascular health of the target population. Notably, there was no correlation between reserve proxies and vascular risk factors measured with the Hachinski’s Ischemic Score in the present study. This suggests that the positive effect of reserve proxies on WML was not mediated by vascular risk factors.

Our results also show that the smaller volumes of WML associated with reserve proxies were also linked to better WM performance than expected for age for the 1-back but not the 2-back condition. This finding supports the contention that proxy-related maintenance of white matter integrity contributes to the preservation of WM function despite older age. Given the known contribution of white matter intergrity to processing speed (Birdsill et al., 2014; Gunning-Dixon & Raz, 2000; Kaplan et al., 2009; Vannorsdall et al., 2009), the protective effect found only for the 1-back condition may be explained by the fact that this condition reflects passive comparison within short-term memory, which may depend on basic cognitive processes such as information processing speed. In turn, preservation of the 2-back performance, which reflects more complex updating processes of WM, may depend on other factors than the sole integrity of white matter tracts. These factors may include cortical thickness in the frontal regions in addition to differences in functional activations or connectivity patterns. The observation that intracranial volume did not moderate the relationship between age and WM indicates that having a larger initial brain volume (as estimated through intracranial volume) does not moderate the effects of age on WM performance, contrary to WML.

The present study has limitations. It relies on a relatively small sample size. As this is a transversal design, the directionality of the association could be reversed as having fewer WML could have enabled individuals to maintain a stimulating lifestyle. Although possible, reversed causality is unlikely because WML generally occurs late in life, whereas composite scores were based on lifelong behavior. Finally, the use of a global weighted reserve proxy score had the advantage of providing a comprehensive indicator of exposure. However, using this proxy prevented establishing which sub-components forming the composite score contributed to the observed effect. An interesting question for future work is to determine more precise proxies that contribute to reserve in older adults.

## Conclusions

This study provides evidence for preserved white matter integrity in older adults, who have had a stimulating lifestyle or lifetime exposure to knowledge, resulting in a smaller effect of age on WM. This indicates that a stimulating lifestyle or lifetime exposure to knowledge helps the brain better resist the accumulation of pathological markers associated with age. This result is consistent with prior findings showing lower amyloid-beta deposition in older people, who have engaged in a more stimulating lifestyle over the course of their lives (Arenaza-Urquijo et al., 2017; Landau et al., 2012). Our study indicates that this protective effect extends to white-matter changes, a phenomenon very common in older adults. Although longitudinal studies with larger sample sizes are needed, the results suggest that differences in lifestyle, socio-behavioural characteristics or exposure to knowledge can have a measurable effect on age-related brain pathology. This study provides support for preventive interventions focused on modifiable risk factors and socio-behavioural characteristics in older adults.

## Supplementary Information

Below is the link to the electronic supplementary material.

Supplementary file1 (DOCX 18 KB)

## Data Availability

Request for data and material can be done by contacting the senior author, Sylvie Belleville.
