# Supplementary material for: Having a stimulating lifestyle is associated with maintenance of white matter integrity with age
Source: Brain Imaging Behav. 2022 Jan 17;16(3):1392–9. doi: 10.1007/s11682-021-00620-7 (PMC9107451; doi:10.1007/s11682-021-00620-7)
Supplement: Supplementary file 1 — Supplementary file1 (DOCX 18 KB) [file 11682_2021_620_MOESM1_ESM.docx]

Supplementary Material

Text S1: Principal Component Analysis Resulting in the Reserve Proxy Composite Score

A principal component analysis (PCA) was used as suggested in (Stern et al., 2005) with the sole purpose of reducing data and creating a single composite score synthesizing multiple proxies of cognitive reserve. This analysis was computed by forcing three proxy measures of cognitive reserve (i.e., scores on the vocabulary subtest and the two CRQ subscales) into a single factor, where each of these proxy measures were weighted to maximize the explained variance. No rotation technique was applied as we forced the three measures into a single factor based on many documented contributions to the concept of “cognitive reserve” in the literature. The resulting Kaiser-Meyer-Olkin index (KMO = 0.592) indicated that partial correlations were sufficiently high to confirm that the data were suitable to perform the analysis, even though part of the data was also expected to be uncorrelated. Thus, there was no minimum load coefficient considered for including a component into the factorial design, and the strict sphericity of the data was not considered as a prerequisite to conducting the analysis (Bartlett sphericity test; *p* = 0.028). The resulting factor accounted for 52.63 % of the common variance, which was considered sufficient given the heterogenous and partly independent nature of the components included.

| **Table S1: Individual Loading Resulting From the Principal Component Analysis for Each Included Component** | |
| --- | --- |
|  | *Loading* |
| Education-Occupation CRQ Subscale | 0.784 |
| Vocabulary Subtest (WAIS-IV) | 0.766 |
| Leisure CRQ Subscale | 0.614 |
